# Study on inpatient expenses and cost control strategies for breast cancer patients based on Diagnosis-Intervention Packet

**DOI:** 10.3389/fpubh.2025.1652174

**Published:** 2025-10-29

**Authors:** Limin Yue, Zhengchen Pan, Wujun Chen

**Affiliations:** Medical Insurance Office, The Affiliated Cancer Hospital of Zhengzhou University & Henan Cancer Hospital, Zhengzhou, China

**Keywords:** medical service, DIP, breast cancer, inpatient expenses, risk factors

## Abstract

**Aim:**

To provide reference information for reducing disease burden and promoting medical service. The inpatient expenses of breast cancer patients in a top-three public hospital in Zhengzhou under the background of Diagnosis-Intervention Packet (DIP) payment reform were analyzed, and corresponding cost control strategies were proposed.

**Methods:**

In this study, 4,590 patients (3,311 before reform and 1,279 after reform) were finally included in this study. Student's *t*-test was used to compare the means of two samples. Chi-square test was used for the comparison of rates. The influencing factors of profit of the patients after reform were analyzed by binary logistic regression.

**Results:**

Post-reform data revealed a significant reduction in medication costs and examination fees (*P* < 0.05), contributing to lower overall hospitalization expenses. The profit ratio of c50.9_86 (c50.9_86.0603: chemotherapy pump implantation surgery for unspecified breast malignant tumors) group was the highest, about 63.2%, and the loss ratio of c50.9_99 (c50.9_99.2503: intravenous injection of chemotherapy drugs for unspecified breast malignant tumors) group was the highest, about 66.7%. The length of hospital stay was the protective factor of profit, whereas the score of disease and employee insurance were the risk factors of profit.

**Conclusion:**

The DIP reform significantly reduced medication costs outside of surgery. Under the DIP payment mode reform policy, hospitals should strengthen the awareness of cost control and reduce medical costs, optimize the score of disease and group setting, pay attention to optimize disease structure and ensure fair access to treatment for patients.

## Introduction

In 2022, breast cancer was the second most common cancer and fourth leading cause of cancer death globally, with an incidence rate in China (33/10,000) far exceeding Southeast Asia's and the highest female cancer mortality rate in the Americas, Europe, and Southeast Asia (1). In China, the annual treatment cost for malignant tumors exceeds 220 billion RMB, which imposes a huge burden on healthcare (2). Since 2009, the growth rate of Chinese basic medical insurance expenditures outpaced that of income by approximately 2.43 percentage points. The swift increase in medical expenditures has placed a heavy burden on the medical insurance fund.

To contain healthcare costs and improve efficiency, China has developed a novel case-based payment system called the Diagnosis-Intervention Packet (DIP), combined with a regional global budget scheme. This reform aligns with the national policy push for a Hierarchical Medical System (HMS), aimed at rationalizing roles and improving efficiency across healthcare institutions (3). Shi et al. (4) reported that the DIP payment reform is more likely to promote the HMS in regions with an advanced policy framework, abundant medical resources, and high-quality primary medical services. Based on the same major diagnosis principles (ICD-10) and similar treatment procedures (ICD9-CM-3), the National Healthcare Security Administration firstly enacted the uniform score catalog and classification standards of the DIP disease groups, containing 11,553 core disease groups and 2,499 comprehensive disease groups (ICD-10 refers to the International Classification of Diseases, Tenth Revision; ICD9-CM-3 refers to the International Classfication of Diseases Clinical Modification of 9th Revision Operations and Procedures). The DIP payment system is a bundled payment model that matches medical services with payment by utilizing historical medical records. In pilot cities, the average score for each DIP disease group was calculated based on the last 3-year average medical cost of inpatient cases. The unit value per score was derived by dividing the regional annual budget by the total annual scores of all inpatient cases, with risk adjustment applied. The payment standard for each group was then determined by multiplying its score by this unit value.

Since its introduction in 2020 across over 70 Chinese cities, the impact of the DIP payment system on inpatient costs remains unclear due to limited evaluation. Among the few existing studies, Lai et al. (5) reported a significant decrease in hospitalization costs and medication expenses after DIP implementation in Guangzhou, whereas Qian et al. (6) observed an increase in the cost of hospitalized patients. There remains a lack of research on the impact of the DIP payment reform on the cost of breast cancer patients. To address this gap, this study focused on Zhengzhou-a major central Chinese city and a DIP pilot city in Henan Province- to examine changes in hospitalization costs for breast cancer patients and evaluate the reform's effect on departmental revenue. Through analysis of health economic indicators, we aim to identify strategies for reducing financial burdens on patients and improving cost containment in hospitals, thereby supporting the optimization and expansion of medical payment reforms.

## Materials and methods

### Data

We collected medical records of breast cancer patients from the top-three public hospital's information system in Zhengzhou between January and December 2023. The inclusion criteria were as follows: (1) primary diagnosis of breast cancer (ICD-10 codes c50, C50.2–5, C50.9). Ensure the homogeneity of the diseases among the research subjects to avoid cost bias caused by differences in diagnosis; (2) age above 18 years, avoid confusion factors caused by age differences; (3) availability of complete clinical records and treatment documentation. Complete data serves as the foundation for conducting cost structure analysis and evaluating policy effectiveness.

Exclusion criteria included: (1) discharge within 24 h without complete clinical data. Short-term hospitalization often indicates mild illness or a diagnostic admission, and its cost structure is not representative; incomplete data make it impossible to accurately classify or conduct cost analysis, which may introduce information bias; (2) presence of severe chronic diseases or complications resulting in hospital stays exceeding 30 days. Extremely long hospital stays are usually accompanied by complex comorbidities or severe complications. The costs are dominated by non-breast cancer factors and do not fall within the scope of typical breast cancer treatment under the DIP payment reform. Excluding these cases can enhance the purity of the cost analysis; (3) cases requiring intensive care or multidisciplinary treatment. The treatment of such patients is highly complex and the resource consumption pattern is particularly unique. Their costs are significantly higher than those of conventional breast cancer patients, and they are not suitable for evaluating the general inpatient cost control strategies based on DIP; (4) incomplete information, transfer to another hospital, death. We collected information on the patient's gender, age, medical insurance type, length of stay, disease coding, disease diagnosis, cost components (total hospitalization, drug, operation, nursing, examination, and inspection expenses). Insufficient information: this prevents the data from being included in the statistical model; Transfer to another hospital: the expenses may be calculated in segments, making it difficult to obtain the full cost. Death: the cost structure of end-of-life care or emergency treatment is abnormal and does not fall within the scope of regular cost analysis.

### Analysis of hospitalization cost index

In the hospital's information system, we identified 12,670 breast cancer patients. According to the inclusion and exclusion criteria, a total of 4,590 patients were included in this study (3,311 before the DIP payment reform and 1,279 after the DIP payment reform). We analyzed changes in hospitalization costs for patients with breast cancer before (January to June 2023) and after (July to December 2023) DIP payment reform. This study would comprehensively consider the purpose of the study and availability of data, and conduct regression analysis on such indicators as gender, age, admission quarter, length of stay, operation or not, disease score, readmission or not, and medical insurance type.

### Statistical analysis

SPSS 26.0 software was used for statistical analysis. The application conditions for Student's *t*-test is: independent samples, homogeneity of variances, and normal distribution. The age, length of stay, and cost of breast cancer patients before and after the DIP payment reform were all independent samples. The data type of the sample were continuous variables. All sample populations are normally distributed and have equal variances. Kolmogorov–Smirnov test: *P* > 0.05. Levene's test: *P* > 0.05. So, Student's *t*-test was used to compare the age, length of stay, and cost of breast cancer patients before and after the DIP payment reform. The Chi-square test is used to compare the rates or proportions of categorical data. Sex, admission quarter, readmission, medical insurance type, profit and loss were categorical data. The comparison of the above indicators between the two groups before and after the DIP payment reform belongs to the comparison of composition ratios, so the Chi-square test was used to analyze where the composition ratio of the above indicators between the two groups. Binary logistic regression is used to study the relationship between multiple independent variables (continuous or categorical) and a binary dependent variable. In this study, profit was a binary classification variable, and the individual variables are mutually independent. There was no collinearity among the continuous independent variables (VIF < 5). Therefore, the influencing factors of profit of the patients after reform were analyzed by binary logistic regression. *P* < 0.05 indicates statistically significant difference.

## Results

### The basic characteristics of the included breast cancer patients

A total of 4,590 patients were included in the final study (3,311 before the reform and 1,279 after the reform). There were no differences in sex, age, admission quarter, length of stay, readmission or not, and medical insurance type between the two groups of patients before and after the DIP payment reform (both *P* > 0.05). The detailed information was shown in Table 1.

**Table 1:** The basic characteristics of the included breast cancer patients.

**Variables**	**Before reform (*n* = 3,311)**	**After reform (*n* = 1,279)**	***t*/χ^2^**	***P-*value**
**Sex**				
Men	11	8	1.925	0.065
Women	3,300	1,271		
Age (years old)	51.41 ± 10.63	51.57 ± 11.95	−0.442	
**Admission quarter**				
No. 1 + No. 2	1,868	744	1.155	0.282
No. 3 + No. 4	1,443	535		
Length of stay (days)	12.67 ± 5.00	12.58 ± 5.18	0.534	0.593
**Readmission**				
No	2,082	782	1.190	0.275
Yes	1,229	497		
**Medical insurance type**				
Resident	1,311	472		0.093
Employee	2,000	807		

### Basic situation and medical expenses of patients with breast cancer

The drug cost and examination cost of the breast cancer cases after the reform were lower than those before the reform (*P* < 0.001). Liu et al. (7) reported that after the DIP payment reform, the proportions of drug cost and examination cost decreased in a tertiary hospital in Guangzhou, which was similar to the present research findings. The operation cost the cases after the reform were higher than that before the reform, as shown in Table 2. There were no differences in total hospitalization cost, nursing cost, and treatment cost between the two groups of patients before and after the DIP payment reform (both *P* > 0.05). A study conducted by Gan et al. (8) also revealed that the operation cost after the DIP payment reform was higher than that before the reform. Qu et al. (9) found that the medical insurance surplus for cases treated with surgery was higher than that for cases treated without surgery. The possible reasons include the following aspects: the DIP payment reform has established a “surplus retention and overspending sharing” mechanism, which prompts hospitals to actively control costs, optimize the treatment process, reduce the use of expensive drugs, and minimize excessive medication and excessive testing behaviors. The essence of the increase in operation cost for inpatients is value reconstruction, which objectively promotes the transformation of medical services from “relying on medicine to support medicine” to “relying on technology to support medicine” and enhances the technical value of doctors (10).

**Table 2:** Basic situation and cost analysis of breast cancer patients ($\bar{x}\pm$s, yuan).

**Variables**	**Before reform (*n* = 3,311)**	**After reform (*n* = 1,279)**	***t***	***P-*value**
Total hospitalization cost	30,633.61 ± 13,122.69	31,019.66 ± 13,851.94	−0.880	0.379
Drug cost	4,594.74 ± 5,420.24	4,123.36 ± 4,748.77	2.731	0.006
Operation cost	5,026.02 ± 3,113.29	5,370.73 ± 3,261.97	−3.318	0.001
Nursing cost	419.11 ± 192.39	429.44 ± 200.38	−1.613	0.107
Treatment cost	1,460.61 ± 1,267.76	1,437.98 ± 1,294.40	0.539	0.590
Examination cost	9,183.81 ± 4,188.42	8,696.70 ± 3,881.59	3.604	< 0.001
Other cost	9,949.32 ± 8,453.97	10,961.45 ± 10,079.74	−3.440	< 0.001

### Profit and loss of DIP disease group

Profit and loss were determined according to the balance of medical insurance. A positive balance was defined as a profit, and a negative balance was defined as a loss (a positive balance means that the medical insurance surplus is positive. A negative balance refers to a situation where the medical insurance account has a deficit). Based on the same major diagnosis principles (ICD-10) and similar treatment procedures (ICD9-CM-3), breast cancer inpatients are classified into five DIP disease groups, such as c50.9_99, c50.9_86, c50.9_85, c50.9_40, and others. The detailed explanation of the “disease groups” can be found in Supplementary Table S1. The profit proportion of the five groups is different (χ^2^ = 11.830, *P* = 0.019). The result was shown in Table 3. The profit proportion of c50.9_86 disease group was the highest, 63.2%, and the loss proportion of c50.9_99 disease group was the highest, 66.7%. The possible reason may be that group c50.9_86 has a high disease score and group c50.9_99 has a low disease score. Some patients in group c50.9_99 have exceeded the limit of medication, such as using expensive Pegylated Recombinant Human Granulocyte Colony Stimulating Factor for Injection beyond medical insurance restrictions.

**Table 3:** Comparison of profit and loss of different disease groups in DIP group [*n* (%)].

**Disease groups**	**Profit [*n* (%)]**	**Loss [*n* (%)]**	**χ^2^**	***P-*value**
c50.9_99	9 (33.3)	18 (66.7)	11.830	0.019
c50.9_86	36 (63.2)	21 (36.8)		
c50.9_85	480 (53.8)	412 (46.2)		
c50.9_40	141 (54.9)	116 (45.1)		
Others	17 (37.0)	29 (63.0)		

### Analysis of factors influencing profit and loss in DIP group

Binary logistic regression analysis was carried out in the DIP group with the loss after reform as the reference group. The results showed that the length of hospital stay is the protective factor of profit, the disease score and the medical insurance type are the risk factors of profit. The probability of profit for patients hospitalized for 5–9, 10–14, and 15 days or more is 0.373 times, 0.334 times, and 0.146 times that of patients hospitalized for less than 5 days, respectively. The probability of profit for employee medical insurance type is 2.786 times that of resident medical insurance. The higher the disease score, the easier it is to make a profit. Sex, age, admission quarter, operation and readmission were not associated with profit in DIP patients (all *P* > 0.05, Table 4). Hosmer–Lemeshow goodness-of-fit test was used to analyze the model fit of logistic regression. The result showed that the model fits well (χ^2^ = 7.021, *P* = 0.534). Gan et al. (8) analyzed the costs of inpatient patients in a tertiary public hospital in Guangzhou under the DIP payment reform. The results showed that the longer the hospital stay, the more likely there was a loss; the higher the disease score, the more likely there was a profit. In line with the present study, Qu et al. (9) also observed a higher medical insurance surplus from employee insurance cases compared to resident insurance cases under the DIP payment reform.

**Table 4:** Results of binary logistic regression analysis of profit and loss factors in DIP group.

**Variables**	**β**	**OR**	**95% CI**	***P*-value**
**Sex (Ref. men)**				
Women	−1.019	0.361	0.040, 3.240	0.363
**Age (years old, Ref**. < **30)**				
30–49	0.341	1.406	0.861, 2.297	0.173
50–69	0.473	1.604	0.981, 2.623	0.060
≥70	0.675	1.965	0.936, 4.124	0.074
**Admission quarter (Ref. No.1)**				
No. 2	−0.208	0.812	0.562, 1.173	0.266
No. 3	−0.007	0.993	0.628, 1.571	0.977
No. 4	−0.329	0.720	0.483, 1.072	0.106
**Length of stay (days, Ref**. < **5)**				
5–9	−0.986	0.373	0.167, 0.832	0.016
10–14	−1.096	0.334	0.148, 0.754	0.008
≥15	−1.927	0.146	0.061, 0.347	< 0.001
**Operation or not (Ref. No)**				
Yes	−1.199	0.302	0.033, 2.728	0.286
Disease score	0.016	1.016	1.014, 1.018	< 0.001
**Readmission (Ref. No)**				
Yes	0.236	1.266	0.944, 1.700	0.116
**Medical insurance type (Ref. Resident)**				
Employee	1.025	2.786	2.067, 3.755	< 0.001

## Discussion

The rapid growth of the inpatient health expenditure poses a serious challenge for the Chinese government (11). Faced with this predicament, the Chinese government has implemented a series of healthcare provider payment reform policies including the DIP payment reform, especially since the National Healthcare Security Administration was established in 2018 (12). We found that the inpatient medical costs per case decreased after the implementation of the DIP payment reform, which was aligned to the related studies (5, 13). During the transition period of payment mode reform, policies and technologies in various aspects are not perfect and mature, and there is a certain impact. The increase of operation cost is mainly due to the optimization and adjustment of the composition of medical service prices due to the reform of DIP payment reform, which indicates that the income of technical labor such as operation and nursing has increased, and the technical value of medical personnel has been recognized (10). It may also be the cause of the use of new medical technology and the severity and particularity of tumor diseases (14–16). Liu et al. (17) reported that after the implementation of the DIP payment reform, the proportion of surgeries at the third and fourth levels has shown an upward trend. In the surgical classification system, surgeries of levels three and four are classified as high-level surgeries. To a certain extent, they can reflect the medical technical level and the ability to handle difficult and complex diseases, and demonstrate the medical level and management decision-making level of the hospital. In this study, the proportion of third and fourth-level surgeries after the DIP payment reform was higher than that before the DIP payment reform (*P* < 0.05). This fully explained the reason for the increase in operation costs after the DIP payment reform. Compared with before the reform, the examination cost has decreased. The possible reason is that hospitals make specific diagnosis and treatment plans for patients according to the severity of their conditions and pay DIP insurance based on them, which can effectively reduce medical expenditure and avoid waste of medical resources (18). The reasons for the increase in other cost compared with that before the reform may be related to the increase in the proportion of patients with difficult and severe diseases treated in top-three hospitals and the increase in the proportion of complex operations, and the development and application of new medical technologies and materials (19). Liu et al. (18) also found that after the implementation of DIP payment reform, the examination cost and other cost for hospitalized patients have significantly decreased compared to before DIP payment reform.

In general, DIP payment reform has positive effects and can effectively reduce the economic burden of hospitalized patients (16). The profit proportion of disease group c50.9_86 was the highest, and the loss proportion of disease group c50.9_99 was the highest. It may be because the disease scores of different DIP groups were different (*F* = 1.219, *P* < 0.001). The disease score of c50.9_86 group were the highest (362.37 ± 65.84), and those of c50.9_99 group were the lowest (169.25 ± 42.96). The logistic regression analysis results of this study showed that the higher the disease score, the easier to make profits. This finding was consistent with the results reported by Li (20), which also indicated that higher disease category scores correlate with greater profitability. For cases with the same diagnosis result, the more difficult the surgical operation is, the higher the score, which reflects the transformation of the current medical reform in China from more work, more pay to better work, better pay, and suggests that medical institutions should control fees to clarify the functional positioning of hospitals and improve the level of treatment, diagnosis and treatment of difficult and complicated cases (21, 22). This can also promote the development of China's classification diagnosis and treatment system to a certain extent. Jiang et al. (23) reported that the longer the length of stay, the lower the profit probability, which was consistent with the present research findings. This is because more hospitalization days will not only generate higher hospitalization costs, but also increase the hospital's medical cost and waste medical resources (24, 25). Therefore, hospital cost control should start from optimizing clinical path and controlling reasonable hospitalization days.

The results of this study showed that accepting urban employee medical insurance patients can easily make hospitals profit, aligning with the findings reported by Jiang et al. (23). The unit price of medical insurance for urban employees is higher than the unit price of medical insurance for urban and rural residents. There is a large difference in the unit price of medical insurance for the two types of medical insurance, which is mainly caused by the difference in the total amount of medical insurance paid by the two types of medical insurance, the number of medical visits and the composition of diseases. Although the score value of each disease is the same, due to the difference in the price of the score value, each disease has different medical insurance funds. Under the same hospital and the same diagnosis and treatment management mode, due to the low price of urban and rural residents' diseases, hospitals will suffer congenital losses in accepting urban and rural residents' patients (26), and hospitals that mainly treat urban and rural residents' patients will also suffer from the phenomenon of “living beyond their means,” and even the situation of shunning urban and rural residents' inpatients. Therefore, it is suggested that when the policy is implemented, the total fund amount of urban and rural employees and urban and rural residents can be balanced, and the principle of the same price for the same disease can be guaranteed. The “unit integral price” and “cross-subsidy” mechanisms can be utilized to promote the balance between the employee and resident medical insurance funds. The specific policy suggestions are as follows.

Deepen payment reform and dynamically adjust the “unit points price”: (i) establish a linkage mechanism for centralized procurement and price reduction: after the centralized procurement and price reduction of drugs and consumables, the scores of relevant disease groups will be synchronously lowered to ensure the sharing of medical insurance surplus and prevent hospitals from exceeding profits (27); and (ii) implement a floating rate mechanism: dynamically adjust DIP point values based on the annual fund budget and total service volume, implement total amount control, and strictly prevent fund bottoming out (28). Establishing a legal “cross subsidy” mechanism to enhance overall resilience: (i) Strengthening government subsidies: Zhou et al. (29) revealed the distribution of government medical subsidies among different medical insurance populations and the important role of fiscal subsidies in resident medical insurance, which is an important reference for the public finance component in the vertical subsidy mechanism; (ii) exploring the family aid of employee medical insurance personal accounts: expand the scope of users of employee medical insurance personal accounts from the employees themselves to their “spouses, parents, children, and close relatives” who participate in basic medical insurance (30). Zhou et al. (31) reported that the average premium paid by employee medical insurance was 5–12 times that of resident medical insurance, which provided an important practical basis for considering the use of the surplus funds of the employee medical insurance to support the resident medical insurance. This structured approach aims to enhance the sustainability and equity of medical insurance system through precise payment reforms and coordinated fiscal mechanisms.

In order to cope with the continuous growth of healthcare expenditures, many countries have carried out similar healthcare payment reform called Diagnosis-related groups (DRGs). Busse et al. (32) conducted a review of DRG payment systems across 12 European countries, noting that such systems contribute to cost containment by establishing predetermined reimbursement rates, as observed in the United States. In European, particularly in England, the implementation of DRGs has promoted a shift toward day-case care, correlating with a general expansion in healthcare service volume. This trend may improve operational adaptability and patient accessibility. Nevertheless, in several European settings, it remains unclear whether the increased service volume elevates overall hospital expenditures. Concurrently, Swedish research indicated that excessive reductions in length of hospital stay could compromise service quality, while studies from other European nations reported no significant changes in mortality or readmission rates following DRG adoption. In alignment with findings from the United States, Switzerland, and Taiwan, research by Lee et al. (33) utilizing extensive claims data from South Korea demonstrated a marked decline in hospitalization duration across various healthcare facilities after DRGs introduction. DRG relies on clinical pathway consensus to form disease groups, with a small number of groups and emphasis on homogeneity; DIP objectively clusters diagnosis and treatment methods based on big data, with a large number of diseases and greater flexibility. DRG and DIP are both reform tools for medical insurance payment methods based on the “prepaid” principle, aimed at controlling the rapid growth of medical expenses and improving the efficiency of medical insurance fund utilization.

### Innovation and limitation

With the deepening of the reform of DIP payment methods, hospitals should conform to the situation, consciously carry out cost control, constantly reduce the proportion of drugs and consumables, and adjust the fee structure, so as to reasonably control medical expenses and make the final cost of patients lower than the DIP score standard. In this way, the hospital obtains both social and economic benefits. In order to better balance economic and social benefits, hospitals should choose diseases that are more valuable to their own development, more representative of the discipline level, with characteristics and core competitiveness or influential on society as advantageous diseases to invest in development. By setting up an audit engine in the intelligent audit system, the hospital can give early warning and prompt to doctors in the process of diagnosis and treatment, and deduct penalties for cases of violation after diagnosis and treatment, so as to realize pre- and post-medical insurance monitoring, reduce the possibility of moral hazard behavior, and promote the sound development of reform.

This study also have some limitations. Firstly, the single-center design of this study, conducted at one academic medical center, limits the generalizability of our findings to hospitals in different settings, with different funding models, or serving different patient populations. Secondly, the short follow-up period at the pilot hospital may not capture long-term effects, as the findings could be influenced by the initial adaptation phase and the natural learning curve associated with the new system. The post-reform cohort was relatively small (1,279 patients compared to 3,311 pre-reform), limiting statistical power. The analysis did not adjust for multiple comparisons between cost categories-increasing the risk of Type I error-nor for potential confounders such as complications, hospital management, or insurance regulatory intensity in the logistic regression. Thirdly, this study has the following conceptual limitations. Terminology of “profit”: the use of “profit” may not align with the regulated nature of hospital finance, potentially skewing interpretations. Adaptation effects: our study provided a static snapshot and may not capture the dynamic strategic adaptations hospitals develop over time in response to financial pressures. Finally, the retrospective, observational design of this study can identify associations but cannot establish causality between the identified factors and hospital profitability, as it relies on historical data with potential biases and cannot firmly establish temporality. Multicenter longitudinal studies can provide more comprehensive evidence from “cause” to “effect.” Therefore, it is necessary to collect longitudinal research data on DIP payment reform data from multiple pilot cities and hospitals with mature DIP operations in the future to further verify the findings of this study.

## Conclusion

China's DIP payment reform has transitioned medical insurance payment from “payment by project” to “payment by disease score” through data-driven, budget constrained, and incentive compatible mechanisms. This shift simultaneously controls costs and guides a systematic change in hospital behavior: hospitals are required to enhance cost-consciousness, promote intelligent auditing, optimize disease structure, and sink simple diseases to grassroots hospitals, thereby improving their capabilities in specialized treatments and overall service quality. Compared with traditional DRG systems, DIP distinguishes itself in three aspects. First, in grouping logic, DIP utilizes big data to naturally form disease combinations from real clinical practice, which is more flexible and can better adapt to clinical diversity and weak medical environments. Second, for budget control, DIP binds the regional total budget with “dynamic point values” to achieve hard constraints and effectively prevent payment risks. Finally, in handling case complexity, DIP accommodates clinical variations through massive grouping, guiding hospitals to focus on process optimization rather than coding manipulation. This model offers a flexible, progressive, and practical path for payment reform in low- and middle-income countries, facilitating optimal allocation of limited medical resources and advancing universal health coverage.

## Data Availability

The raw data supporting the conclusions of this article will be made available by the authors, without undue reservation.
