# Supplementary material for: Study on inpatient expenses and cost control strategies for breast cancer patients based on Diagnosis-Intervention Packet
Source: Front Public Health. 2025 Oct 29;13:1652174. doi: 10.3389/fpubh.2025.1652174 (PMC12605485; doi:10.3389/fpubh.2025.1652174)
Supplement: Supplementary file 1 [file Table_1.DOCX]

Supplementary Table 1 A plain-language interpretation of disease group

| Disease groups | interpretation |
| --- | --- |
| c50.9_99 | c50.9_99.2503 (Intravenous injection of chemotherapy drugs for unspecified breast malignant tumors) |
| c50.9_86 | c50.9_86.0603 (Chemotherapy pump implantation surgery for unspecified breast malignant tumors) |
| c50.9_85 | c50.9_85.4500×001 (Unilateral radical mastectomy with ipsilateral axillary sentinel lymph node biopsy for unspecified breast malignant tumors);  c50.9_85.4500 (Unilateral radical mastectomy for unspecified malignant breast tumors);  c50.9_85.4401 (Bilateral breast modified radical mastectomy for unspecified breast malignant tumors);  c50.9_85.4303 (Unilateral simple mastectomy with regional lymph node dissection for unspecified breast malignant tumors);  c50.9_85.4302 (Unilateral breast conserving modified radical mastectomy for unspecified breast malignant tumors);  c50.9_85.4301 (Unilateral modified radical mastectomy for unspecified breast malignant tumors);  c50.9_85.4300×003 (Unilateral mastectomy with ipsilateral axillary lymph node biopsy for unspecified breast malignant tumors);  c50.9_85.4100×001 (Unilateral mastectomy for unspecified malignant breast tumors);  c50.9_85.3300×001 (Unilateral mastectomy with implant placement for unspecified malignant breast tumors);  c50.9_85.2300×001 (Localized breast enlargement resection for unspecified breast malignant tumors);  c50.9_85.2101 (Minimally invasive rotational resection for unspecified breast malignant tumors);  c50.9_85.2100×019 (Breast gland segmentectomy for unspecified malignant breast tumors);  c50.9_85.2100×003 (Breast lesion excision surgery for unspecified breast malignant tumor);  c50.9_85.1200×001 (Breast biopsy procedure for unspecified breast malignant tumor);  c50.9_85.1100×001 (Mammary gland puncture biopsy for unspecified breast malignant tumor); |
| c50.9_40 | c50.9_40.1105,85.4302 (Sentinel lymph node biopsy, unilateral breast conserving modified radical mastectomy for unspecified breast malignant tumor);  c50.9_40.1105,85.4100×001 (Sentinel lymph node biopsy, unilateral mastectomy for unspecified breast malignant tumor);  c50.9_40.1105,85.1100×001,85.4302 (Sentinel lymph node biopsy, Mammary gland puncture biopsy, unilateral breast conserving modified radical mastectomy for unspecified breast malignant tumor);  c50.9_40.1105 (Sentinel lymph node biopsy for unspecified breast malignant tumor);  c50.9_40.1103 (Axillary lymph node biopsy for unspecified breast malignant tumor);  c50.9_40.1102 (Organ biopsy of supraclavicular lymph nodes for unspecified breast malignant tumor); |
| Others | c50.9_ (Unspecified breast malignant tumor);  c50_3_3 (Related surgeries for breast malignant tumor);  c50_3_2 (Related surgeries for breast malignant tumor);  c50_3_1 (Related surgeries for breast malignant tumor);  c50_2_3 (Therapeutic operation for breast malignant tumor);  c50_2_2 (Therapeutic operation for breast malignant tumor);  c50_2_1 (Therapeutic operation for breast malignant tumor);  c50_1_-1,0,1,2,3 (Diagnostic operation for breast malignant tumor) |
